# Associated factors and short-term mortality of early versus late acute kidney injury following on-pump cardiac surgery

**DOI:** 10.1093/icvts/ivac118

**Published:** 2022-05-16

**Authors:** Shengnan Li, Ming Liu, Xiang Liu, Dong Yang, Nianguo Dong, Fei Li

**Affiliations:** 1 Department of Anesthesiology, Union Hospital, Tongji Medical College, Huazhong University of Science and Technology, Wuhan, China; 2 Institute of Anesthesiology and Critical Care Medicine, Union Hospital, Tongji Medical College, Huazhong University of Science and Technology, Wuhan, China; 3 Department of Cardiovascular Surgery, Union Hospital, Tongji Medical College, Huazhong University of Science and Technology, Wuhan, China; 4 Guangzhou AID Cloud Technology Co., LTD, Guangzhou, China

**Keywords:** Acute kidney injury, Cardiac surgery, Early AKI, Late AKI, Risk factors

## Abstract

**OBJECTIVES:**

Acute kidney injury (AKI) is common following cardiac surgery. The aim was to investigate the characteristics of AKI that occurred within 48 h and during 48 h to 7 days after cardiac surgery.

**METHODS:**

Patient data were extracted from Medical Information Mart for Intensive Care III database. AKI was defined according to the Kidney Disease Improving Global Outcomes guideline and divided into early (within 48 h) and late (during 48 h to 7 days) AKI. Multivariable logistic regression models were established to investigate risk factors for AKI. Cox proportional hazards model was used to analyse 90-day survival.

**RESULTS:**

AKI occurred in 51.2% (2741/5356) patients within the first 7 days following cardiac surgery, with the peak occurrence at 36–48 h. The incidence of early and late AKI was 41.9% and 9.2%, respectively. Patients with late AKI were older and had more comorbidities compared to early AKI patients. Risk factors associated with early AKI included age, body mass index, congestive heart failure and diabetes. While late AKI was related to atrial fibrillation, estimated glomerular filtration rate, sepsis, norepinephrine, mechanical ventilation and packed red blood cell transfusion. In Cox proportional model, both late and early AKIs were independently associated with 90-day mortality, and patients with early AKI had better survival than those with late AKI.

**CONCLUSIONS:**

AKI that occurred earlier was distinguishable from AKI that occurred later after cardiac surgery. Time frame should be taken into consideration.

**Figure:**
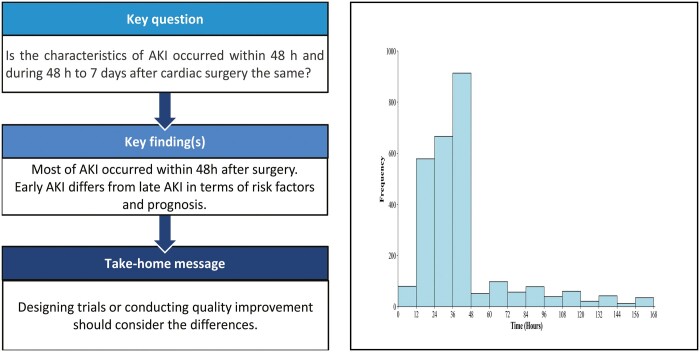

## INTRODUCTION

Acute kidney injury (AKI) is common following cardiac surgery and has been tied to prolonged hospitalization, higher economic cost, increased readmissions and poor prognosis [1–3]. Incidence of AKI following cardiac surgery is reported to range from 0.3% to 81.2% according to different definitions as well as different population [4, 5]. Mild AKI, even small increment of serum creatine (sCr) or transient oliguria has been reported to be associated with poor prognosis [6, 7]. Furthermore, previous studies have also shown that AKI, even with complete recovery, is still a risk factor for long-term mortality [8, 9].

During cardiac surgery, cardiopulmonary bypass is a well-known deleterious factor to kidney, for the process itself could lead to potential hypo-perfusion, hypoxia and/or ischaemic–reperfusion, oxidative stress and exacerbate inflammation [10]. While some postoperative complications or therapeutic measurements, like low cardiac output, sepsis, use of vasoactive agents or antibiotics, may also hurt kidney by specific mechanisms [1]. Although several models have been established to focus on risk factors or prediction of AKI following cardiac surgery, seldom of them take time frame into consideration, and try to distinguish AKI occurring earlier from that occurring later. Recently, AKI following non-cardiac major surgery has been shown to be 2 different phenotypes by occurring time [11].

In this retrospective database analysis, we sought to compare risk factors between patients with AKI occurring within 48 h post-cardiac surgery (called early AKI) and patients with AKI occurring during 48 h to 7 days post-cardiac surgery (called late AKI). We also evaluated their effects on 90-day mortality.

## PATIENTS AND METHODS

### Ethics statement

Medical Information Mart for Intensive Care (MIMIC) III v1.4 database was approved by the Institutional Review Boardsof Beth Israel Deaconess Medical Center (number 2001-P-001699/14) and the Massachusetts Institute of Technology (number 0403000206). Requirements for individual patient consent were waived. Shengnan Li obtained access to this public database (certification number 36216625) and signed data use agreement including not to identify subjects. Institutional Review Boards approval for this records-based research was exempted according to Common Rule (45 CFR 46).

### Data source

We conducted a retrospective analysis based on MIMIC III v1.4 database, which is a restricted-access clinical database hosted on Physionet. It includes 61 532 intensive care unit (ICU) stays admitted into 5 ICUs at Beth Israel Deaconess Medical Center from June 2001 to October 2012 [12–14]. The database includes demographics, vital sign measurement, laboratory test results, procedures, medications, caregiver notes, imaging reports and mortality. Health-related data are de-identified in compliance with Health Insurance Portability and Accountability Act. Protected health information was also deleted from the structured data source.

### Patient population

Adult patients undergoing on-pump cardiac surgery were included in this study. Exclusive criteria were (i) patient who was lack of sCr, urine output and renal replacement therapy (RRT) information and (ii) patient had end stage of renal disease or estimated glomerular filtration rate (eGFR) <15 ml/min, or patient was on RRT before surgery. For those who was performed >1 surgery during the time interval, we only selected the first surgery. International Classification of Diseases Version 9 (ICD-9) code was used to identify surgical procedures and extracted data on demographics, health characteristics, lab test data, postoperative complications and treatments. Surgery was divided into 5 categories: coronary artery bypass grafting, valve surgery, aorta surgery, combined surgery and others. Here, we defined combined surgery as >1 procedure was done during the surgery.

### Outcomes

Primary outcome was AKI. AKI was defined according to Kidney Disease Improving Global Outcomes (KDIGO) rule by sCr criteria or urine output criteria [15]. Baseline sCr was defined in the same way as a previous study [11]. ‘Early AKI’ was defined as AKI firstly diagnosed within 48 h following surgery. ‘Late AKI’ was defined as AKI firstly diagnosed during 48 h to 7 days after surgery. Secondary outcomes included 90-day survival, length of ICU stay and length of hospital stay. Patient survival following discharge was contained in the database, which was acquired from social security death registry.

### Statistical analysis

For a detailed description, see Supplementary Material. Continuous variables were presented with mean (standard deviation) or median (interquartile range), while categorical variables were presented with frequency (%). Normality was accessed by Skewness/Kurtosis test. Missing data were imputed using the multiple imputation by chained equation [16]. Baseline characteristics were compared among groups using Kruskal–Wallis test or analysis of variance (ANOVA) for continuous variables and chi-squared test or Fisher’s exact test for categorical variables. Survival model was initiated at the time of surgery and followed until death or last following up. Survival curve were constructed by Kaplan–Meier method, and pairwise log-rank test was used to test difference in survival. Bonferroni correction was used to offset multiple comparisons. Multivariable Cox’s proportional hazard model was constructed to compare hazards ratio across the groups (no AKI, early AKI, late AKI) adjusting by potential factors that may affect survival. Two multivariable logistic regression models were established to investigate independent risk factors for early AKI and late AKI, respectively (both compared with patients who did not develop AKI).

For the machine learning algorithm, a copy of data was split into 70% training set for model development and 30% testing set for validation in a stratified fashion. Two separate fine-tuned random forest models were developed using 2 sets of features from above to classify early AKI patients and late AKI patients (both versus patients without AKI). The feature selection pipeline in these 2 models was both based on univariate feature selection and random-forest based recursive feature selection. Trained models were validated on the testing set. Discrimination was evaluated by area under the receiver operating characteristic curve (AUC), and calibration was assessed by Hosmer–Lemeshow test. Statistical analysis was performed with Python 3.7.5 under anaconda environment (http://www.anaconda.com/). *P*-value <0.05 was considered statistically significant.

## RESULTS

### Baseline characteristics of patients with early AKI, late AKI or without AKI

**Figure 1:**
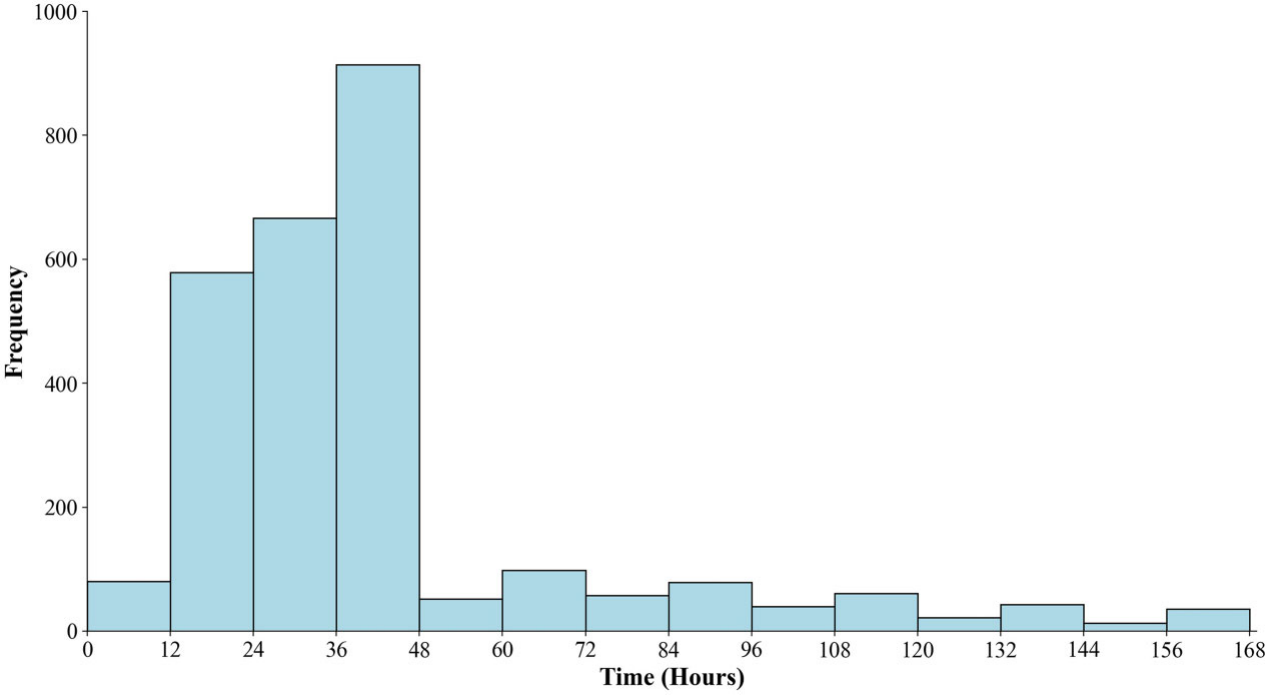
Time distribution of acute kidney injury frequency following on-pump cardiac surgery.

There were 5356 patients in the final dataset (Supplementary Material, Fig. S1, flow chart). AKI occurred in 51.2% (*n* = 2741) patients within the first 7 days following cardiac surgery (Table 1 and Fig. 1). The peak occurring time for AKI was 36–48 h following cardiac surgery (Fig. 1). Most (81.9%, *n* = 2246) of them occurred within the first 48 h post-surgery, defined as early AKI. And less (18.1%, *n* = 495) occurred during 48 h to 7 days post-surgery, defined as late AKI (Table 1 and Fig. 1). Then, we looked into severity distribution of early and late AKI separately (Supplementary Material, Tables S1 and S2). In early AKI population, there were 51.2% (*n* = 1149), 45.7% (*n* = 1027) and 3.1% (*n* = 70) for KDIGO stage 1, stage 2 and stage 3, respectively (Supplementary Material, Table S1). In late AKI population, there were 74.5% (*n* = 369), 22.4% (*n* = 111), and 3.0% (*n* = 15) for KDIGO stage 1, stage 2 and stage 3, respectively (Supplementary Material, Table 2). Patients with early AKI [0.97 (0.36) mg/dl] had similar baseline sCr level to patients with late AKI [1.04 (0.43) mg/dl], and both of them were within the normal range. However, patients with late AKI were older, more likely to combine with atrial fibrillation, congestive heart failure and chronic kidney disease compared to early AKI patients. They also had higher rate in taking medications, like angiotensin-converting enzyme inhibitor/angiotensin II receptor blocker, calcium channel blocker, as well as higher probability of using epinephrine and norepinephrine. Late AKI patients were more likely to had lower cardiac index (<2.2 l/min m^2^), longer mechanical ventilation duration and more red blood cell transfusion (Table 1).

**Table 1:** Baseline characteristics of patients undergoing cardiac surgery

Variable	No AKI (*n* = 2615)	Early AKI (*n* = 2246)	Late AKI (*n* = 495)	*P*-Value^*^	*P*-Value^**^
Age, years	65.6 (12.0)	66.8 (11.6)	69.3 (11.6)	<0.001	<0.001
Male	1819 (69.6%)	1552 (69.1%)	331 (66.9%)	0.493	
BMI, kg/m^2^	27.6 (4.9)	29.9 (5.8)	27.5 (4.9)	<0.001	<0.001
eGFR, ml/min/1.73 m^2^	88.1 (72.1, 99.1)	85.6 (66.7, 97.7)	80.3 (58.4, 95.6)	<0.001	<0.001
Reference sCr, mg/dl	0.93 (0.29)	0.97 (0.36)	1.04 (0.43)	<0.001	<0.001
Admission type				<0.001	0.002
Emergent	1248 (47.7%)	1110 (49.4%)	287 (58.0%)		
Urgent	84 (3.2%)	96 (4.3%)	21 (4.2%)		
Elective	1283 (49.1%)	1040 (46.3%)	187 (37.8%)		
Race				0.116	
White	1873 (71.6%)	1608 (71.6%)	334 (67.5%)		
Black	82 (3.1%)	57 (2.5%)	11 (2.2%)		
Other	660 (25.2%)	581 (25.9%)	150 (30.3%)		
Atrial fibrillation	993 (38.0%)	982 (43.7%)	271 (54.8%)	<0.001	<0.001
Congestive heart failure	554 (21.2%)	656 (29.2%)	171 (34.6%)	<0.001	0.022
Coronary heart disease	1969 (75.3%)	1775 (79.0%)	394 (79.6%)	0.004	0.826
Diabetes	722 (27.6%)	818 (36.4%)	162 (32.7%)	<0.001	0.134
Hypertension	1747 (66.8%)	1459 (65.0%)	312 (63.0%)	0.171	
Cerebrovascular disease	18 (0.7%)	23 (1.0%)	6 (1.2%)	0.322	
Chronic liver disease	38 (1.5%)	29 (1.3%)	8 (1.6%)	0.813	
CKD	92 (3.5%)	122 (5.4%)	44 (8.9%)	<0.001	0.005
COPD	39 (1.5%)	37 (1.7%)	13 (2.6%)	0.193	
Multicomorbidities	2101 (80.3%)	1922 (85.6%)	437 (88.3%)	<0.001	0.133
Sofa score	4.6 (2.3)	4.7 (2.5)	5.1 (2.8)	0.001	0.001
ACE inhibitor	846 (32.4%)	708 (31.5%)	192 (38.8%)	0.007	0.002
ARB	123 (4.7%)	115 (5.1%)	38 (7.7%)	0.023	0.033
CCB	247 (9.5%)	192 (8.6%)	63 (12.7%)	0.015	0.005
Beta-blocker	2081 (79.6%)	1565 (69.7%)	358 (72.3%)	<0.001	0.267
Furosemide	2038 (77.9)	1550 (69.0%)	348 (70.3%)	<0.001	0.61
Statins	1673 (64.0%)	1298 (57.8%)	302 (61.0%)	<0.001	0.206
Aspirin	2297 (87.8%)	1817 (80.9%)	407 (82.2%)	<0.001	0.537
Acetaminophen	2343 (89.6%)	1874 (83.4%)	417 (84.2%)	<0.001	0.711
Ibuprofen	149 (5.7%)	91 (4.1%)	19 (3.8%)	0.016	0.926
Vasopressin	64 (2.5%)	101 (4.5%)	31 (6.3%)	<0.001	0.122
Dobutamine	21 (0.8%)	36 (1.6%)	10 (2.0%)	0.012	0.645
Epinephrine	364 (13.9%)	375 (16.7%)	120 (24.2%)	<0.001	<0.001
Norepinephrine	172 (6.6%)	231 (10.3%)	83 (16.8%)	<0.001	<0.001
Cardiac index^a^	1700 (65.0%)	1573 (70.0%)	376 (76.0%)	<0.001	0.01
CVP >14cm H_2_O	359 (13.7%)	406 (18.1%)	79 (16.0%)	<0.001	0.293
IABP	109 (4.2%)	213 (9.5%)	56 (11.3%)	<0.001	0.248
MV, h	5.5 (3.6, 13)	6.8 (3.9, 17.3)	11.6 (5.0, 22.0)	<0.001	<0.001
Sepsis	117 (4.5%)	209 (9.3%)	64 (12.9%)	<0.001	0.019
PRBC transfusion, ml	211.3 (475.6)	320.6 (678.9)	532.9 (943.9)	<0.001	<0.001
Surgery type				<0.001	0.004
CABG	1466 (56.1%)	1238 (55.1%)	240 (48.5%)		
Valve	637 (24.4%)	521 (23.2%)	109 (22.0%)		
Aorta	4 (0.2%)	5 (0.2%)	2 (0.4%)		
Other	42 (1.6%)	26 (1.2%)	5 (1.0%)		
Combined	466 (17.8%)	456 (20.3%)	139 (28.1%)		
Haemoglobin, g/dl	11.2 (2.3)	11.1 (2.1)	11.1 (2.1)	0.031	
Platelet, 10^9^/l	193.1 (68.5)	196.9 (69.1)	192.3 (68.7)	0.106	
INR	1.3 (0.3)	1.4 (0.3)	1.4 (0.4)	0.001	0.001
ALT, IU/l	32.1 (23.3)	33.2 (27.4)	31.3 (30.1)	0.078	
AST, IU/l	44.2 (33.2)	46.1 (37.0)	46.6 (37.7)	0.503	
BUN, mg/dl	17.6 (7.2)	19.5 (8.6)	20.9 (10.8)	<0.001	<0.001
Ca^+^, mg/dl	8.6 (0.7)	8.6 (0.6)	8.6 (0.7)	0.781	
Glucose, mg/dl	123.5 (36.8)	126.9 (39.6)	130.2 (45.6)	<0.001	<0.001
Lactate, mmol/l	1.9 (1.1)	2.0 (1.2)	2.0 (1.2)	0.198	
Potassium, mEq/l	4.2 (0.5)	4.3 (0.5)	4.3 (0.5)	0.006	0.006
Na^+^, mEq/l	139.2 (2.8)	139.2 (3.0)	139.4 (3.4)	0.249	
BP-Art, mmHg	78.6 (15.4)	78.3 (15.7)	78.8 (18.1)	0.557	
Bilirubin, mg/dl	0.7 (0.4)	0.7 (0.4)	0.7 (0.5)	0.329	

Continuous variables were presented in mean (SD) or median (IQR), and categorical variables were presented in frequency (%).

a Cardiac index <2.2 l/min m^2^.

* *P*-value for comparison among the 3 groups.

** *P*-value for comparison between early and late AKI.

ACE: angiotensin-converting enzyme; AKI: acute kidney injury; ALT: alanine aminotransferase; ARB: angiotensin II receptor blocker; AST: aspartate transaminase; BMI: body mass index; BP-Art: atrial blood pressure; BUN: blood urea nitrogen; CABG: coronary artery bypass graft; CCB: calcium channel blocker; CKD: chronic kidney disease; COPD: chronic obstruct pulmonary disease; CVP: central venous pressure; eGFR: estimated glomerular filtration rate; IABP: intra-aortic balloon pump; INR: international standard ratio; IQR: interquartile range; MV: mechanical ventilation; PRBC: packed red blood cell; sCr: serum creatine; SD: standard deviation.

**Table 2:** Multivariable logistic regression models for early and late acute kidney injury

Variables	Early AKI (*n* = 5356)			Late AKI (*n* = 3110)		
	OR	95% CI	*P*-Value	OR	95% CI	*P*-Value
Age (10 years)	1.09	1.03–1.16	0.004			
BMI (kg/m^2^)	1.09	1.08–1.11	<0.001			
Atrial fibrillation				1.46	1.16–1.84	0.001
Congestive heart failure	1.29	1.13–1.48	<0.001			
Diabetes	1.18	1.04–1.34	0.009			
Beta-blocker	0.76	0.65–0.88	0.001			
Furosemide	0.73	0.63–0.85	<0.001			
Haemoglobin,^a^ max–min	0.91	0.88–0.94	<0.001			
Potassium,^a^ max–min	1.19	1.06–1.32	0.002			
eGFR^b^						
60–90				1.03	0.79–1.33	0.849
<60				1.69	1.23–2.33	0.001
Surgery type^c^						
Valve				1.32	0.86–2.05	0.205
Aorta				1.99	0.29–13.5	0.483
Other				1.43	0.49–4.16	0.507
Combined				1.49	1.13–1.96	0.005
Sepsis				1.53	1.04–2.24	0.029
IABP				1.58	1.04–2.39	0.032
Norepinephrine				1.48	1.05–2.09	0.026
Mechanical ventilation (12 h)				1.10	1.05–1.16	<0.001
PRBC transfusion				1.14	1.07–1.21	<0.001

Two multivariable logistic regression models were established, and both of them were compared with patients without AKI. For early AKI model, we only included preoperative variables for selection, while we included both preoperative and postoperative variables in late AKI model.

a Difference between maximum and minimum haemoglobin/potassium value within 1 month prior to hospital admission.

b Referred to eGRF >90 ml/min/1.73 m^2^.

c Referred to CABG surgery.

AKI: acute kidney injury; BMI: body mass index; CABG: coronary artery bypass graft; CI: confidence interval; eGFR: estimated glomerular filtration rate; IABP: intra-aortic balloon pump; OR: odds ratio; PRBC: packed red blood cell.

### Risk factors for patients with early AKI or late AKI

To investigate risk factors for early and late AKI, we established 2 separate multivariable logistic regression models. Early and late AKI were set as outcomes. Univariable analysis was presented in Supplementary Material, Tables S4 and S5. Independent risk factors were different in these 2 subgroups (Table 2). Early AKI was more relevant with basic health conditions like age, body mass index, congestive heart failure, diabetes and fluctuation of potassium 1 month prior to hospital admission. Beta-blocker and furosemide were protective for early AKI. While late AKI was mainly associated with atrial fibrillation, decreased renal reserve function (eGFR < 60 ml/min) as well as postoperative factors, like sepsis, mechanical ventilation duration, packed red blood cell transfusion, usage of norepinephrine and intro-aortic balloon pump.

### Prediction of early AKI and late AKI by machine learning

**Figure 2:**
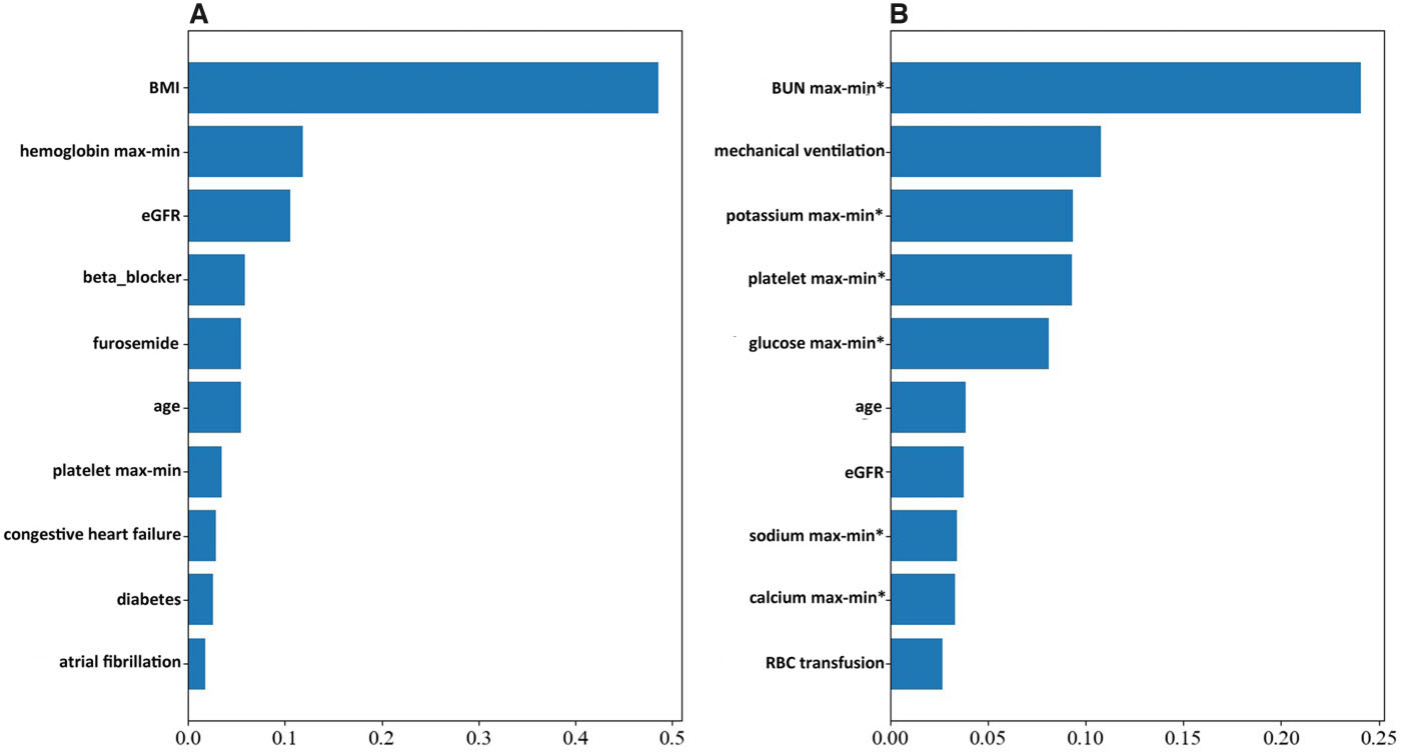
Variable importance plot of the random forest model. (**A**) Random forest model to predict early acute kidney injury versus no acute kidney injury. (**B**) Random forest model to predict late acute kidney injury versus no acute kidney injury. Max–min: difference between maximum and minimum value within one month prior to hospital admission. Max–min*: difference between maximum and minimum value during hospitalization.

We predicted early and late AKI by random forest models. Importance of covariates in the final models was presented (Fig. 2). Again, variable spectra for early and late AKIs were different (Fig. 2). In the training dataset, AUC [95% confidence interval (CI)] for late AKI was 0.76 (0.72, 0.79), while AUC (95% CI) for early AKI was 0.67 (0.65, 0.70) (Supplementary Material, Fig. S2). However, both models showed suboptimal calibration as per the Hosmer–Lemeshow test, *P *<* *0.001 (Supplementary Material, Fig. S3). In the testing dataset, AUC (95% CI) for late AKI was 0.75 (0.73, 0.78) and 0.64 (0.62, 0.66) for early AKI.

### Prognosis of patients with early AKI or late AKI

**Figure 3:**
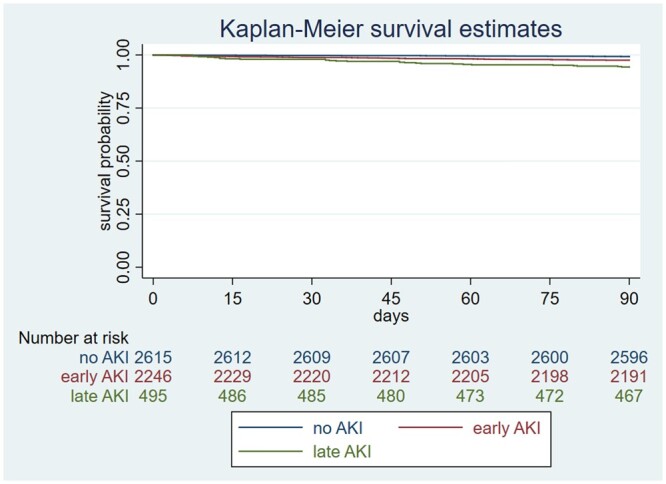
90-Day survival curve for patients undergoing cardiac surgery. Pairwise log-rank test was used to test difference in survival. Bonferroni correction were used to offset multiple comparisons. Early acute kidney injury versus no acute kidney injury: *P *<* *0.001; late acute kidney injury versus no acute kidney injury: *P *<* *0.001; early acute kidney injury versus late acute kidney injury: *P *<* *0.001.

Patients with late AKI had worse prognosis compared to those with early AKI (Table 3). They had both longer hospital stay [9.97 (7.22–13.97) vs 7.86 (5.69–11.36) days, *P *<* *0.001] and ICU stay [3.48 (2.11–6.12) vs 2.32 (1.3–4.16) days, *P *<* *0.001] compared to patients with early AKI (Table 3). They also had higher 90-day mortality compared to patients with early AKI as well (Table 3 and Fig. 3). In Cox proportional hazard model for patient’s mortality, both early and late AKIs remained independent predictors for mortality after adjustment. Late AKI showed a stronger association with 90-day mortality, with hazards ratio: 4.40, 95% CI: 2.42–8.02, *P *<* *0.001, while hazards ratio [95% CI] for early AKI was 2.69 [1.57–4.61], *P *<* *0.001 (Supplementary Material, Table S6 and Table 4).

**Table 3:** Outcomes for early and late acute kidney injury

	No AKI	Early AKI	Late AKI	*P*-Value^*^	*P-*Value^**^
LOS-ICU (days)	1.9 (1.2, 2.9)	2.3 (1.3, 4.2)	3.5 (2.1, 6.1)	<0.001	<0.001
LOS hospital (days)	6.9 (5.2, 9.1)	7.9 (5.7, 11.4)	10.0 (7.2, 14.0)	<0.001	<0.001
Mortality_90 days	19 (0.7%)	55 (2.4%)	28 (5.6%)	<0.001	<0.001

Continuous variables were presented in median (IQR), and categorical variables were presented in frequency (%).

* *P*-value for comparison among no AKI, early AKI and late AKI.

** *P*-value for comparison between early and late AKI.

AKI: acute kidney injury; IQR: interquartile range; LOS hospital: length of hospital stay; LOS-ICU: length of ICU stay.

**Table 4:** Cox proportional hazard model for mortality following hospital discharge

Parameter (reference level)	Level	Adjusted HR	95% CI	*P*-Value
No AKI	Early AKI	2.69	1.57-4.61	<0.001
	Late AKI	4.40	2.42-8.02	<0.001

AKI was stratified by no AKI, early AKI and late AKI. No AKI was set as reference, and adjusted by age, gender, BMI, eGFR, atrial fibrillation, congestive heart failure, hypertension, chronic liver disease, COPD, sofa score, sepsis, IABP and cardiac index.

AKI: acute kidney injury; BMI: body mass index; CI: confidence interval; COPD: chronic obstruct pulmonary disease; eGFR: estimated glomerular filtration rate; HR: hazard ratio; IABP: intra-aortic balloon pump; .

## DISCUSSION

In this retrospective study, most of the AKI manifested within 48 h postoperatively. We classified AKI following cardiac surgery into early AKI (within 48 h post-surgery) and late AKI (during 48 h to 7 days post-surgery) deliberately. Our major results confirm similar findings with a previous study that early AKI and late AKI following surgery may be 2 different phenotypes [11]. In the present study, most of the risk factors appear to be specific to patients with early AKI or late AKI. Furthermore, the effects of early and late AKI on 90-day mortality were also distinguishable. These findings may add extra evidence that AKI occurring after cardiac surgery may also need to be treated separately according to its onset, especially when design clinical trials.

Definition of AKI has changed over time, and it mainly relies on sCr, urine output or RRT. AKI has been defined from 0.1 mg/dl increment in sCr to RRT in the previous literature [5]. Thus, incidence of AKI varies [5]. Here, we define AKI according to KIDGO rule by both sCr or urine output criteria. And incidence of AKI was 51.2% in our population, which was consistent with recent reports that defined AKI according to the same criteria by recent data [4, 17, 18].

AKI following cardiac surgery has been discussed previously. Predictive models have been established to predict severe AKI [19–25]. Some of them have great performance [19–24]. However, very few studies focused on the onset of AKI following cardiac surgery. Acute Dialysis Quality Initiative used to advocate subdividing cardiac surgery associated AKI into early AKI (within the 7 days) and late AKI (between 7 and 30 days after cardiac surgery) based on median length of hospital stay [26]. They suggested that it might be necessary to discriminate cardiac surgery induced AKI and cardiac surgery associated AKI [26]. Another literature defined early AKI as that occurred within 24 h following cardiac surgery by RIFLE criteria when they explored attributable cardiopulmonary bypass-related variables on AKI [27]. Among critically ill patients following non-cardiac major surgery, 48 h was set as cut-off to define early and late AKI [11]. However, when we looked into the occurring time distribution of AKI after cardiac surgery, the peak was 36–48 h, and 81.9% of AKI occurred within 48 h. Thus, we identified 48 h as a reasonable watershed to separate early AKI and late AKI. This time window is also consistent with the plausible delay of serum creatinine until it reaches diagnostic criteria and codified in KDIGO definition of AKI. As predicted, random forest model for late AKI showed better discrimination performance than early AKI model, for the former was more informative.

Risk factors for AKI after cardiac surgery have been demonstrated before. Variables like age, body mass index, congestive heart failure and diabetes have been frequently mentioned as risk factors for AKI post-cardiac surgery previously [19, 20, 23, 28]. However, they affected only early AKI in our study. Medications like beta-blocker and furosemide were also only related to early AKI rather than late AKI. Decreased eGFR is a component in simplified renal index score system [21], but it only had impact on late AKI. Compared to coronary artery bypass grafting surgery, combined surgery has been reported as a significant risk factor for AKI after cardiac surgery [20, 21]. However, it was a risk factor only for late AKI in our study. Moreover, surgery type did not have relative effect on early AKI. Considering decreased eGFR and a series of postoperative complications, occurrence of late AKI is more likely due to ‘long-term’ accumulation of repeated insults, which is also confirmed by focused analysis on the incidence of AKI among patients with multiple cardiac surgeries. Thus, it seems that it is necessary to take time frame into consideration when evaluating risk for AKI following cardiac surgery. Different predictive or evaluating systems should be established for early and late AKI, respectively.

Here, we also found some modifiable factors. Anaemia has been recognized modifiable in previous studies [28–30]. In the present study, the difference between maximum and minimum preoperative haemoglobin was protective for early AKI, while packed red blood cell transfusion was a risk factor for late AKI. Usually, if haemoglobin value was normal, it would be relatively stable and would not be interfered too much before surgery. A significant increment in haemoglobin before surgery may be explained by alleviating anaemia. It seemed that we added indirect but significant evidence on the benefit of anaemia improvement before surgery. Demirjian *etal.* had demonstrated that serum potassium was a risk factor for postoperative dialysis [23]. In our study, fluctuation of potassium 1 month before hospital admission was a risk factor for early AKI. It is usual for cardiac patients to take both diuretics and potassium supplements. Congestive heart failure might damage potassium absorption from gastrointestinal tract. Diuretic itself could result in loss of potassium. Significant fluctuation of potassium may be reflection of poorly controlled cardiac disease. Carefully monitoring and maintaining potassium level normal should be helpful.

### Limitations

Our study also has some limitations. First, it was a retrospective study and, thus, had its inherent nature. We established the association between independent risk factors and AKI, but not causality. However, if carefully validated, it could help clinicians to identify patients with high risks and take preventive steps to improve patient care. Second, our data came from a single centre. However, we studied a relatively large population. We had complete data to define AKI stage with precise information on timing. Third, we did not include intraoperative variables, which were also very important for outcomes. It was unfortunate that this information was not available in our dataset. However, we had reasonably comprehensive preoperative and postoperative variables, which could be reasonably expected to impact AKI rate.

## CONCLUSION

In summary, we classified AKI following on-pump cardiac surgery into 2 categories, early AKI and late AKI according to the occurring time. Furthermore, we identified both common and distinct risk factors for early and late AKIs. They had different risk factor spectrum and prognosis. Therefore, it may need to treat the situations distinctively when designing trials or conducting quality improvement.

## SUPPLEMENTARY MATERIAL

Supplementary material is available at *ICVTS* online.

**Conflict of interest:** none declared.

## Data Availability Statement

Dataset analysed in this study is available from the corresponding author. However, reanalysis of the full data or for other use should be approved from the MIMIC Institute.

## Author contributions

**Shengnan Li:** Conceptualization; Data curation; Investigation; Methodology; Resources; Validation; Writing—original draft. **Ming Liu:** Methodology; Validation; Writing—review & editing. **Xiang Liu:** Data curation; Formal analysis; Methodology; Software; Validation; Writing—review & editing. **Dong Yang:** Data curation; Formal analysis; Methodology; Software; Visualization; Writing—original draft. **Nianguo Dong:** Conceptualization; Investigation; Supervision; Validation; Writing—review & editing. **Fei Li:** Conceptualization; Data curation; Investigation; Methodology; Supervision; Validation; Writing—review & editing.

## Reviewer information

Interactive CardioVascular and Thoracic Surgery thanks Stefano Schena and the other, anonymous reviewer(s) for their contribution to the peer review process of this article.

## Supplementary Material

ivac118_Supplementary_Data

## ABBREVIATIONS

AKI: Acute kidney injury
AUC: Area under the receiver operating characteristic curve
CI: Confidence interval
eGFR: Estimated glomerular filtration rate
ICU: Intensive care unit
KDIGO: Kidney Disease Improving Global Outcomes
MIMIC: Medical Information Mart for Intensive Care
RRT: Renal replacement therapy
sCr: Serum creatine
