# Supplementary material for: Associated factors and short-term mortality of early versus late acute kidney injury following on-pump cardiac surgery
Source: Interact Cardiovasc Thorac Surg. 2022 May 16;35(3):ivac118. doi: 10.1093/icvts/ivac118 (PMC9419684; doi:10.1093/icvts/ivac118)
Supplement: ivac118_Supplementary_Data [file ivac118_supplementary_data.zip › ivac118_Supplementary_Data/ivac118_Supplementary_Material.docx]

**Logistic regression models**

Two separate multivariable logistic regression models were established, and both of them were compared with patients without AKI. In early AKI model, we only included preoperative variables for selection, while we included both preoperative and postoperative variables in late AKI model. First, univariable logistic regression models were used to identify relationship between perioperative variables and early or late AKI. Second, variables with *P*<0.05 in univariable analysis were entered into multivariable logistic regression models by forward stepwise to identify independent influence on early or late AKI. Collinearity diagnostic tests were used to exclude variables that could lead to multicollinearity. Model comparisons was by Akaike Information Criterion minimization. Only variables with *P*<0.05 in univariable analysis were presented.

**Survival analysis**

Survival information was analyzed by Kaplan-Meier method, and comparison between groups were performed by log-rank test. Bonferroni correction were used to offset multiple comparisons. Multivariable Cox’s proportional hazard model was constructed to compare hazards ratio across the groups (no AKI, early AKI, late AKI) adjusting by potential factors that may affect survival. Variables with *P*<0.05 in univariable analysis were presented and were entered into final Cox proportional hazard model. Proportional hazard assumption was verified by Schoenfeld residuals.

**Machine learning**

We predicted early AKI and late AKI by two separate random forest models. A copy of data was split into 70% training set and 30% testing set in stratified fashion. Data in training set was used to develop models, and data in testing set was used to validate. Two separate fine-tuned random forest models were developed using two sets of features to classify early AKI patients and late AKI patients (both versus patients without AKI). Random forest model is not a fixed model, and multiple hyper-parameters should be tuned by applying model tunning techniques, such as grid search method. Here, grid search together with 5-fold cross validation method were used to tune the random forest model. The hyper-parameters of models were searched from the following scope:

- max_feature: [5,6,7,8]
- min_sample_leaf: [10,20,50,80,110,150]
- max_depth: [3,5,8,10]
- n_estimater: [200,400,600,800,1000]
- min_sample_split: [2,3,5,7]
- criterion: [‘gini’,’entropy’]

The hyperparameters of the best models presented as follows:

|  | early AKI model | late AKI model |
| --- | --- | --- |
| max_feature | 5 | 6 |
| min_sample_leaf | 110 | 10 |
| max_depth | 8 | 3 |
| n_estimater | 1000 | 400 |
| min_sample_split | 2 | 2 |
| criterion | ‘gini’ | ‘gini’ |

Features in final models were determined by recursive feature elimination (RFE). RFE is a feature selection method that fits a model and removes the weakest feature (or features) until the specified number of features is reached. In each step, features are ranked by the model’s coefficient or feature importance. By recursively eliminating a small number of features per loop, RFE attempts to eliminate dependencies and collinearity that may exist in the model. Here, we fit a random forest model in every step of the feature eliminating loop. Performance of final trained models were assessed by AUC. Goodness of fit were evaluated by Homser-lemeshow test. Final trained models were validated on testing set. Discrimination on testing set were tested by AUC.
