# Supplementary material for: Associated factors and short-term mortality of early versus late acute kidney injury following on-pump cardiac surgery
Source: Interact Cardiovasc Thorac Surg. 2022 May 16;35(3):ivac118. doi: 10.1093/icvts/ivac118 (PMC9419684; doi:10.1093/icvts/ivac118)
Supplement: ivac118_Supplementary_Data [file ivac118_supplementary_data.zip › ivac118_Supplementary_Data/ivac118_Supplementary_Tables S1_to_S6-Figures_S1_to_S2.docx]

**Supplemental Table S1**. Baseline characteristics of patients undergoing cardiac surgery classified by early AKI stages

| Variables | No AKI (n=3110) | Stage 1 (n=1149) | Stage 2 (n=1027) | Stage 3 (n=70) | p-value |
| --- | --- | --- | --- | --- | --- |
| Age | 66.2(12.0) | 66.7(11.7) | 66.8(11.5) | 67.3(11.2) | 0.611 |
| Male | 2150(69.1%) | 797(69.4%) | 715(69.6%) | 40(57.1%) | 0.183 |
| BMI | 27.6(4.9) | 28.9(5.3) | 31.1(6.1) | 31.2(6.9) | <0.001 |
| eGFR | 87.3(70.1,98.5) | 86.8(71.1,98.3) | 82.6(62.7,96.4) | 89.3(65.6,101.7) | <0.001 |
| Reference sCr | 0.95(0.32) | 0.94(0.32) | 1.02(0.38) | 0.97(0.52) | <0.001 |
| Admission type |  |  |  |  | 0.018 |
| Emergent | 1535(49.4%) | 536(46.7%) | 535(52.1%) | 39(55.7%) |  |
| Urgent | 105(3.4%) | 42(3.7%) | 51(5.0%) | 3(4.3%) |  |
| Elective | 1470(47.3%) | 571(49.7%) | 441(42.9%) | 28(40.0%) |  |
| Race |  |  |  |  | 0.118 |
| White | 2207(71.0%) | 830(72.2%) | 719(70.0%) | 59(84.3%) |  |
| Black | 93(3.0%) | 23(2.0%) | 33(3.2%) | 1(1.4%) |  |
| Other | 810(26.1%) | 296(25.8%) | 275(26.8%) | 10(14.3%) |  |
| Atrial fibrillation | 1264(40.6%) | 482(42.0%) | 469(45.7%) | 31(44.3%) | 0.043 |
| Congestive heart failure | 725(23.3%) | 297(25.9%) | 330(32.1%) | 29(41.4%) | <0.001 |
| Coronary heart disease | 2363(76.0%) | 911(79.3%) | 815(79.4%) | 49(70.0%) | 0.017 |
| Diabetes | 884(28.4%) | 382(33.3%) | 406(39.5%) | 30(42.9%) | <0.001 |
| Hypertension | 2059(66.2%) | 752(65.5%) | 670(65.2%) | 37(52.9%) | 0.133 |
| Cerebrovascular disease | 24(0.8%) | 10(0.9%) | 11(1.1%) | 2(2.9%) | 0.262 |
| Chronic liver disease | 46(1.5%) | 10(0.9%) | 19(1.9%) | 0(0%) | 0.174 |
| CKD | 136(4.4%) | 55(4.8%) | 63(6.1%) | 4(5.7%) | 0.148 |
| COPD | 52(1.7%) | 23(2.0%) | 13(1.3%) | 1(1.4%) | 0.61 |
| Multicomorbidities | 2538(81.6%) | 970(84.4%) | 892(86.9%) | 60(85.7%) | 0.001 |
| ACE inhibitor | 1038(33.4%) | 342(29.8%) | 340(33.1%) | 26(37.1%) | 0.12 |
| ARB | 161(5.2%) | 61(5.3%) | 49(4.8%) | 5(7.1%) | 0.817 |
| CCB | 310(10.0%) | 95(8.3%) | 89(8.7%) | 8(11.4%) | 0.273 |
| Beta blocker | 2439(78.4%) | 852(74.2%) | 666(64.9%) | 47(67.1%) | <0.001 |
| Furosemide | 2386(76.7%) | 875(76.2%) | 631(61.4%) | 44(62.9%) | <0.001 |
| Statins | 1975(63.5%) | 704(61.3%) | 554(53.9%) | 40(57.1%) | <0.001 |
| Aspirin | 2704(87.0%) | 973(84.7%) | 787(76.6%) | 57(81.4%) | <0.001 |
| Acetaminophen | 2760(88.8%) | 1003(87.3%) | 812(79.1%) | 59(84.3%) | <0.001 |
| Ibuprofen | 168(5.4%) | 43(3.7%) | 45(4.4%) | 3(4.3%) | 0.13 |
| Vasopressin | 95(3.1%) | 33(2.9%) | 58(5.7%) | 10(14.3%) | <0.001 |
| Dobutamine | 31(1.0%) | 15(1.3%) | 18(1.8%) | 3(4.3%) | 0.03 |
| Epinephrine | 484(15.6%) | 200(17.4%) | 160(15.6%) | 15(21.4%) | 0.285 |
| Norepinephrine | 255(8.2%) | 99(8.6%) | 119(11.6%) | 13(18.6%) | <0.001 |
| Cardiac index^#^ | 2076(66.8%) | 801(69.7%) | 725(70.6%) | 47(67.1%) | 0.074 |
| CVP>14 | 438(14.1%) | 187(16.3%) | 201(19.6%) | 18(25.7%) | <0.001 |
| IABP | 165(5.3%) | 107(9.3%) | 100(9.7%) | 6(8.6%) | <0.001 |
| MV | 6.0(3.8,14.6) | 6.2(3.8,16.8) | 7.1(4.0,17.3) | 10.6(4.0,23.5) | <0.001 |
| Sepsis | 181(5.8%) | 68(5.9%) | 129(12.6%) | 12(17.1%) | <0.001 |
| RBC transfusion | 262.4(587.9) | 269.5(571.0) | 362.7(737.9) | 540.9(1163.5) | <0.001 |
| Surgery type |  |  |  |  | 0.678 |
| CABG | 1706(54.9%) | 647(56.3%) | 552(53.8%) | 39(55.7%) |  |
| Valve | 746(24.0%) | 259(22.5%) | 240(23.4%) | 22(31.4%) |  |
| Aorta | 6(0.2%) | 3(0.3%) | 2(0.2%) | 0(0%) |  |
| Other | 47(1.5%) | 14(1.2%) | 11(1.1%) | 1(1.4%) |  |
| Combined | 605(19.5%) | 226(19.7%) | 222(21.6%) | 8(11.43%) |  |
| Hemoglobin | 11.2(2.2) | 11.1(2.1) | 11.1(2.1) | 11.3(2.1) | 0.52 |
| Platelet | 193.0(68.5) | 194.6(69.2) | 200.0(69.5) | 190.5(60.0) | 0.043 |
| INR | 1.3(0.3) | 1.4(0.3) | 1.4(0.3) | 1.4(0.3) | 0.121 |
| ALT | 31.9(24.5) | 31.7(24.5) | 35.0(30.3) | 31.9(25.2) | 0.032 |
| AST | 44.5(33.9) | 43.7(34.4) | 48.6(40.0) | 49.0(31.1) | 0.016 |
| BUN | 18.1(8.0) | 18.6(7.7) | 20.5(9.3) | 20.9(9.7) | <0.001 |
| Ca^+^ | 8.6(0.7) | 8.6(0.6) | 8.5(0.6) | 8.7(0.7) | 0.08 |
| Glucose | 124.6(38.4) | 124.9(37.9) | 128.7(41.2) | 132.6(42.2) | 0.001 |
| lactate | 1.9(1.1) | 2.0(1.2) | 2.1(1.3) | 2.1(1.6) | 0.099 |
| potassium | 4.2(0.5) | 4.3(0.5) | 4.3(0.5) | 4.3(0.6) | 0.017 |
| Na^+^ | 139.2(2.9) | 139.3(3.0) | 139.1(3.0) | 138.3(3.5) | 0.286 |
| BP-Art | 78.6(15.9) | 78.5(17.0) | 78.1(14.2) | 76.7(15.2) | 0.554 |
| Bilirubin | 0.7(0.4) | 0.7(0.4) | 0.7(0.4) | 0.7(0.6) | 0.668 |
| Hemoglobin* | 11.5(2.3) | 11.4(2.2) | 11.4(2.2) | 11.3(2.0) | 0.09 |
| Platelet* | 199.3(68.1) | 200.3(68.4) | 205.0(70.2) | 197.8(64.0) | 0.226 |
| INR* | 1.3(0.3) | 1.3(0.3) | 1.3(0.3) | 1.3(0.3) | 0.056 |
| ALT* | 31.5(23.3) | 31.5(23.0) | 34.0(28.3) | 29.9(26.0) | 0.063 |
| AST* | 38.1(25.6) | 39.2(30.4) | 42.4(34.5) | 41.0(24.7) | 0.126 |
| BUN* | 18.6(8.2) | 18.8(7.7) | 20.7(9.4) | 20.6(9.6) | <0.001 |
| Ca^+^* | 8.6(0.7) | 8.6(0.6) | 8.6(0.6) | 8.7(0.7) | 0.462 |
| Glucose* | 127.2(40.7) | 127.3(41.7) | 134.6(48.6) | 140.1(39.8) | <0.001 |
| Lactate* | 1.8(1.0) | 1.8(1.1) | 1.9(1.1) | 2.0(1.5) | 0.007 |
| Potassium* | 4.2(0.5) | 4.2(0.6) | 4.3(0.6) | 4.2(0.7) | 0.065 |
| Na^+^* | 139.0(3.0) | 139.2(3.1) | 139.0(3.2) | 138.0(4.1) | 0.104 |
| Bilirubin* | 0.7(0.4) | 0.7(0.4) | 0.7(0.4) | 0.7(0.6) | 0.763 |

CKD: chronic kidney disease, COPD: chronic obstruct pulmonary disease, ACE inhibitor: Angiotensin-converting enzyme (ACE) inhibitor, ARB: Angiotensin ii receptor blocker, CCB: calcium channel blocker, IABP: intra-aortic balloon pump, MV: mechanical ventilation duration (hour), RBC transfusion: red blood cell transfusion (ml), CABG: coronary artery bypass grafting, INR: international standard ratio, ALT: alanine aminotransferase, AST: aspartate transaminase, BUN: blood urea nitrogen, BP-Art: atrial blood pressure, ^#^Cardiac index < 2.2 L/min·m^2^. *value within one month prior to hospital admission. Continuous variables were presented in mean (sd) or median (IQR), and categorical variables were presented in frequency (percent).

**Supplemental Table S2.** Baseline characteristics of patients undergoing cardiac surgery classified by late AKI stages

| Variables | No AKI (n=2615) | Stage 1 (n=369) | Stage 2 (n=111) | Stage 3 (n=15) | p-value |
| --- | --- | --- | --- | --- | --- |
| Age | 65.6(12.0) | 68.6(11.9) | 71.4(10.1) | 69.6(11.4) | <0.001 |
| Male | 1819(69.6%) | 247(66.9%) | 73(65.8%) | 11(73.3%) | 0.621 |
| BMI | 27.6(4.9) | 27.5(4.7) | 27.9(5.5) | 27.2(5.6) | 0.955 |
| eGFR | 88.1(72.1,99.1) | 80.3(56.6,96.3) | 77.0(59.8,90.5) | 83.8(72.7,94.1) | <0.001 |
| Reference sCr | 0.93(0.29) | 1.05(0.45) | 1.03(0.35) | 0.96(0.26) | <0.001 |
| Admission type |  |  |  |  | <0.001 |
| Emergent | 1248(47.7%) | 212(57.5%) | 67(60.4%) | 8(53.3%) |  |
| Urgent | 84(3.2%) | 14(3.8%) | 7(6.3%) | 0(0%) |  |
| Elective | 1283(49.1%) | 143(38.8%) | 37(33.3%) | 7(46.7%) |  |
| Race |  |  |  |  | 0.173 |
| White | 1873(71.6%) | 247(66.9%) | 78(70.3%) | 9(60.0%) |  |
| Black | 82(3.1%) | 7(1.9%) | 4(3.6%) | 0(0%) |  |
| Other | 660(25.2%) | 115(31.2%) | 29(26.1%) | 6(40.0%) |  |
| Atrial fibrillation | 993(38.0%) | 205(55.6%) | 58(52.3%) | 8(53.3%) | <0.001 |
| Congestive heart failure | 554(21.2%) | 120(32.5%) | 48(43.2%) | 3(20.0%) | <0.001 |
| Coronary heart disease | 1969(75.3%) | 298(80.8%) | 86(77.5%) | 10(66.7%) | 0.105 |
| Diabetes | 722(27.6%) | 125(33.9%) | 32(28.8%) | 5(33.3%) | 0.093 |
| Hypertension | 1747(66.8%) | 239(64.8%) | 65(58.6%) | 8(53.3%) | 0.189 |
| Cerebrovascular disease | 18(0.7%) | 4(1.1%) | 2(1.8%) | 0(0.0%) | 0.501 |
| Chronic liver disease | 38(1.5%) | 6(1.6%) | 2(1.8%) | 0(0%) | 0.946 |
| CKD | 92(3.5%) | 33(8.9%) | 10(9.0%) | 1(6.7%) | <0.001 |
| COPD | 39(1.5%) | 11(3.0%) | 2(1.8%) | 0(0.0%) | 0.201 |
| Multicomorbidities | 2101(80.3%) | 326(88.4%) | 99(89.2%) | 12(80.0%) | <0.001 |
| ACE inhibitor | 846(32.4%) | 137(37.1%) | 50(45.1%) | 5(33.3%) | 0.016 |
| ARB | 123(4.7%) | 33(8.9%) | 5(4.5%) | 0(0%) | 0.005 |
| CCB | 247(9.5%) | 51(13.8%) | 11(9.9%) | 1(6.7%) | 0.069 |
| Beta blocker | 2081(79.6%) | 274(74.3%) | 73(65.8%) | 11(73.3%) | 0.001 |
| Furosemide | 2038(77.9%) | 271(73.4%) | 69(62.2%) | 8(53.3%) | <0.001 |
| Statins | 1673(64.0%) | 233(63.1%) | 60(54.1%) | 9(60.0%) | 0.201 |
| Aspirin | 2297(87.8%) | 314(85.1%) | 81(73.0%) | 12(80.0%) | <0.001 |
| Acetaminophen | 2343(89.6%) | 322(87.3%) | 84(75.7%) | 11(73.3%) | <0.001 |
| Ibuprofen | 149(5.7%) | 13(3.5%) | 5(4.5%) | 1(6.7%) | 0.359 |
| Vasopressin | 64(2.5%) | 22(6.0%) | 8(7.2%) | 1(6.7%) | <0.001 |
| Dobutamine | 21(0.8%) | 6(1.63%) | 3(2.7%) | 1(6.7%) | 0.014 |
| Epinephrine | 364(13.9%) | 84(22.8%) | 33(29.7%) | 3(20.0%) | <0.001 |
| Norepinephrine | 172(6.6%) | 60(16.3%) | 21(18.9%) | 2(13.3%) | <0.001 |
| Cardiac index^#^ | 1700(65.0%) | 273(74%) | 92(82.9%) | 11(73.3) | <0.001 |
| CVP>14 | 359(13.7%) | 55(14.9%) | 20(18.0%) | 4(26.7%) | 0.277 |
| IABP | 109(4.2%) | 37(10.0%) | 15(13.5%) | 4(26.7%) | <0.001 |
| MV | 5.5(3.6,13.0) | 11.1(5.0,21.5) | 12.5(5.1,24.6) | 14.8(6.4,30.0) | <0.001 |
| Sepsis | 117(4.5%) | 43(11.7%) | 19(17.1%) | 2(13.3%) | <0.001 |
| RBC transfusion | 211.3(475.6) | 507.6(946.0) | 598.8(887.7) | 667.3(1284.8) | <0.001 |
| Surgery type |  |  |  |  | <0.001 |
| CABG | 1466(56.1%) | 179(48.5%) | 53(47.8%) | 8(53.3%) |  |
| Valve | 637(24.4%) | 79(21.4%) | 27(24.3%) | 3(20.0%) |  |
| Aorta | 4(0.2%) | 1(0.3%) | 0(0%) | 1(6.7%) |  |
| Other | 42(1.6%) | 4(1.1%) | 1(0.9%) | 0(0%) |  |
| Combined | 466(17.8%) | 106(28.7%) | 30(27.0%) | 3(20.0%) |  |
| Hemoglobin | 11.2(2.3) | 11.1(2.1) | 11.3(2.2) | 11.1(2.2) | 0.687 |
| Platelet | 193.1(68.5) | 189.7(67.8) | 199.8(72.8) | 202.5(59.2) | 0.577 |
| INR | 1.3(0.3) | 1.4(0.4) | 1.4(0.4) | 1.3(0.2) | 0.017 |
| ALT | 32.1(23.3) | 30.3(23.7) | 34.1(46.2) | 35.7(17.3) | 0.053 |
| AST | 44.2(33.2) | 46.6(37.6) | 45.8(39.2) | 51.0(30.1) | 0.462 |
| BUN | 17.6(7.2) | 20.7(10.7) | 21.3(10.4) | 22.8(14.8) | <0.001 |
| Ca^+^ | 8.6(0.7) | 8.6(0.7) | 8.7(0.6) | 8.4(0.5) | 0.166 |
| Glucose | 123.5(36.8) | 128.6(40.9) | 131.9(55.7) | 155.6(64.6) | 0.041 |
| lactate | 1.9(1.1) | 2.1(1.3) | 1.8(0.9) | 1.7(0.6) | 0.492 |
| potassium | 4.2(0.5) | 4.3(0.5) | 4.2(0.5) | 4.3(0.3) | 0.698 |
| Na^+^ | 139.2(2.8) | 139.4(3.3) | 139.4(3.4) | 139.3(3.6) | 0.37 |
| BP-Art | 78.6(15.4) | 78.4(19.0) | 79.2(15.2) | 86.6(13.9) | 0.156 |
| Bilirubin | 0.7(0.4) | 0.7(0.4) | 0.8(0.4) | 1.2(1.0) | 0.003 |
| Hemoglobin* | 11.5(2.3) | 11.5(2.1) | 11.5(2.2) | 11.2(2.1) | 0.9 |
| Platelet* | 199.4(68.1) | 196.6(68.5) | 205.1(67.9) | 204.3(61.1) | 0.759 |
| INR* | 1.3(0.3) | 1.4(0.4) | 1.4(0.4) | 1.4(0.3) | 0.03 |
| ALT* | 31.6(22.5) | 29.4(23.8) | 34.2(36.7) | 35.0(17.6) | 0.006 |
| AST* | 37.6(24.4) | 38.9(26.1) | 44.7(45.0) | 43.4(19.7) | 0.35 |
| BUN* | 18.1(7.4) | 21.4(11.0) | 21.5(10.3) | 24.0(14.1) | <0.001 |
| Ca^+^* | 8.6(0.7) | 8.6(0.7) | 8.7(0.7) | 8.4(0.6) | 0.418 |
| Glucose* | 126.0(39.0) | 132.2(43.3) | 135.1(59.2) | 154.9(67.5) | 0.026 |
| Lactate* | 1.7(0.9) | 1.9(1.3) | 1.8(0.8) | 2.0(0.7) | 0.026 |
| Potassium* | 4.2(0.5) | 4.3(0.6) | 4.2(0.6) | 4.3(0.4) | 0.672 |
| Na^+^* | 139.0(3.0) | 139.1(3.4) | 139.4(3.4) | 138.3(3.7) | 0.286 |
| Bilirubin* | 0.7(0.4) | 0.7(0.4) | 0.7(0.4) | 0.8(0.4) | 0.169 |

CKD: chronic kidney disease, COPD: chronic obstruct pulmonary disease, ACE inhibitor: Angiotensin-converting enzyme (ACE) inhibitor, ARB: Angiotensin ii receptor blocker, CCB: calcium channel blocker, IABP: intra-aortic balloon pump, MV: mechanical ventilation duration (hour), RBC transfusion: packed red blood cell transfusion (ml), CABG: coronary artery bypass grafting, INR: international standard ratio, ALT: alanine aminotransferase, AST: aspartate transaminase, BUN: blood urea nitrogen, BP-Art: atrial blood pressure, ^#^Cardiac index < 2.2 L/min·m^2^, *value at hospital admission. Continuous variables were presented in mean (sd) or median (IQR), and categorical variables were presented in frequency (percent).

**Supplemental Table S3**. Lab test characteristics of patients undergoing cardiac surgery

|  | No AKI (n=2615) | Early AKI (n=2246) | Late AKI (n=495) | p-value^*^ | | p-value^**^ | |
| --- | --- | --- | --- | --- | --- | --- | --- |
| Hemoglobin max-min | 2.8(1.3,4.3) | 2.3(1.0,3.8) | 2.7(1.2,4.0) | | <0.001 | | <0.001 |
| platelet max-min | 67.0(34.0,108.0) | 64.0(30.1,104.0) | 69.9(32.0,113.0) | | 0.02 | | 0.02 |
| INR max-min | 0.3(0.1,0.5) | 0.3(0.1,0.5) | 0.3(0.1,0.6) | | 0.001 | | 0.001 |
| ALT max-min | 3.0(0,17.0) | 3.7(0,17.0) | 2.6(0,15.1) | | 0.11 | |  |
| AST max-min | 6.0(0,31.4) | 6.4(0,36.5) | 5.0(0,31.4) | | 0.11 | |  |
| BUN max-min | 4.0(2.0,8.0) | 4.0(1.0,7.43) | 5.0(2.0,10.0) | | <0.001 | | <0.001 |
| Ca max-min | 0.3(0.0,0.8) | 0.4(0.0,0.8) | 0.3(0.0,0.7) | | 0.735 | |  |
| Glucose max-min | 22.0(0.0,55.0) | 25.0(0.0,58.0) | 29.0(0.0,72.0) | | <0.001 | | <0.001 |
| Lactate max-min | 1.28(0.6,2.1) | 1.2(0.5,2.2) | 1.4(0.5,2.3) | | 0.439 | |  |
| Potassium max-min | 0.5(0.1,0.9) | 0.5(0.1,1.0) | 0.6(0.2,1.1) | | 0.005 | | 0.005 |
| Sodium max-min | 3.0(0.0,5.0) | 2.0(0.0,5.0) | 3.0(0.4,5.0) | | 0.03 | | 0.03 |
| Bilirubin max-min | 0.1(0.0,0.34) | 0.1(0.0,0.3) | 0.1(0.0,0.4) | | 0.747 | |  |

INR: international standard ratio, ALT: alanine aminotransferase, AST: aspartate transaminase, BUN: blood urea nitrogen. Max-min: difference between maximum and minimum value within one month prior to hospital admission. Continuous variables were presented in mean (sd) or median (IQR). **p*-value for comparison among the three groups. ***p*-value for comparison between early and late AKI.

Supplementary Table S4. Univariable and multivariable logistic regression model of early AKI

| Variables | Univariable | | | Multivariable | | | | |
| --- | --- | --- | --- | --- | --- | --- | --- | --- |
|  | OR | 95% CI | *P*-value | | OR | 95%CI | *P*-value | |
| Admission type |  |  | <0.001 | |  |  | |  |
| Urgent | 1.29 | 0.97-1.72 | 0.081 | | 1.11 | 0.82-1.52 | | 0.495 |
| Emergent | 1.02 | 0.91-1.14 | 0.699 | | 1.06 | 0.92-1.22 | | 0.391 |
| eGFR^&^ |  |  | 0.002 | |  |  | |  |
| 60-90 | 1.04 | 0.92-1.17 | 0.501 | | 0.95 | 0.83-1.09 | | 0.467 |
| <60 | 1.34 | 1.15-1.57 | <0.001 | | 1.12 | 0.94-1.35 | | 0.213 |
| Age (10 year) | 1.04 | 1.00-1.09 | 0.062 | | 1.09 | 1.03-1.16 | | **0.004** |
| BMI (kg/m^2^) | 1.09 | 1.08-1.10 | <0.001 | | 1.09 | 1.08-1.10 | | **<0.001** |
| Ventricular fibrillation | 1.72 | 1.09-2.72 | 0.019 | | 1.57 | 0.97-2.54 | | 0.064 |
| Atrial fibrillation | 1.13 | 1.02-1.27 | 0.024 | | 1.11 | 0.98-1.26 | | 0.101 |
| Congestive heart failure | 1.36 | 1.20-1.54 | <0.001 | | 1.29 | 1.13-1.48 | | **<0.001** |
| Coronary heart disease | 1.19 | 1.05-1.36 | 0.009 | | 1.03 | 0.89-1.20 | | 0.660 |
| Diabetes | 1.44 | 1.28-1.62 | <0.001 | | 1.18 | 1.04-1.34 | | **0.009** |
| Beta blocker | 0.63 | 0.56-0.72 | <0.001 | | 0.76 | 0.65-0.88 | | **<0.001** |
| Furosemide | 0.68 | 0.60-0.76 | <0.001 | | 0.73 | 0.63-0.85 | | **<0.001** |
| Calcium channel blocker | 0.84 | 0.70-1.02 | 0.079 | | 0.84 | 0.69-1.03 | | 0.102 |
| Hemoglobin max-min* | 0.91 | 0.88-0.93 | <0.001 | | 0.91 | 0.88-0.94 | | **<0.001** |
| Potassium max-min* | 1.08 | 0.98-1.19 | 0.102 | | 1.19 | 1.06-1.32 | | **0.002** |
| AST max-min* | 1.00 | 1.00-1.00 | 0.002 | | 1.00 | 1.00-1.00 | | **0.007** |

OR, Odds Ratio; CI, confidence interval; eGFR, estimated glomerular infiltration rate; BMI, body mass index; AST aspartate transaminase. & referred to eGRF> 90 ml/min/1.73 m^2^. * difference between maximum and minimum hemoglobin/potassium/AST value within one month prior to hospital admission. All preoperative variables as well as surgical information were included in univariable logistic regression model. Only variables with *P*<0.05 in univariable analysis were presented in table and were entered into multivariable analysis. Variables with *P*<0.05 after adjustment were shown as bold.

Supplementary Table S5. Univariable and Multivariable logistic regression model of late AKI

| Variables | Univariable | | | | Multivariable | | |
| --- | --- | --- | --- | --- | --- | --- | --- |
|  | OR | | 95%CI | *P*-value | OR | 95%CI | *P*-value |
| Admission type* |  |  | | <0.001 |  |  |  |
| Urgent | 1.72 | 1.04-2.83 | | 0.035 | 0.92 | 0.51-1.63 | 0.768 |
| Emergent | 1.58 | 1.29-1.93 | | <0.001 | 1.15 | 0.90-1.47 | 0.277 |
| Ethnicity** |  |  | | 0.045 |  |  |  |
| black | 0.75 | 0.40-1.43 | | 0.383 | 0.77 | 0.39-1.54 | 0.46 |
| other | 1.27 | 1.03-1.58 | | 0.025 | 1.26 | 0.99-1.59 | 0.053 |
| eGFR*** |  |  | | <0.001 |  |  |  |
| 60-90 | 1.40 | 1.12-1.75 | | 0.003 | 1.03 | 0.79-1.33 | 0.849 |
| <60 | 2.95 | 2.27-3.83 | | <0.001 | 1.69 | 1.23-2.33 | **0.001** |
| Surgery type**** |  |  | | <0.001 |  |  |  |
| Valve surgery | 1.05 | 0.82-1.34 | | 0.723 | 1.32 | 0.86-2.05 | 0.205 |
| Aorta surgery | 3.05 | 0.56-16.8 | | 0.199 | 1.99 | 0.29-13.5 | 0.483 |
| Other surgery | 0.72 | 0.28-1.86 | | 0.505 | 1.43 | 0.49-4.16 | 0.507 |
| Combined surgery | 1.82 | 1.44-2.30 | | <0.001 | 1.49 | 1.13-1.96 | **0.005** |
| Age (10year) | 1.31 | 1.21-1.43 | | <0.001 | 1.09 | 0.97-1.21 | 0.142 |
| Atrial fibrillation | 1.98 | 1.63-2.40 | | <0.001 | 1.46 | 1.16-1.84 | **0.001** |
| Congestive heart failure | 1.96 | 1.60-2.42 | | <0.001 | 1.10 | 0.85-1.42 | 0.456 |
| Coronary heart disease | 1.28 | 1.01-1.62 | | 0.04 | 1.23 | 0.79-1.90 | 0.352 |
| Diabetes | 1.28 | 1.04-1.57 | | 0.021 | 1.23 | 0.98-1.56 | 0.079 |
| Multicomorbidities | 1.84 | 1.38-2.47 | | <0.001 | 1.03 | 0.71-1.50 | 0.886 |
| Angiotensin receptor blocker | 1.68 | 1.16-2.46 | | 0.007 | 1.40 | 0.93-2.10 | 0.111 |
| Beta blocker | 0.67 | 0.54-0.83 | | <0.001 | 0.78 | 0.59-1.03 | 0.079 |
| Furosemide | 0.67 | 0.54-0.83 | | <0.001 | 0.80 | 0.60-1.05 | 0.107 |
| INR max-min^#^ | 1.63 | 1.30-2.03 | | <0.001 | 0.90 | 0.69-1.20 | 0.459 |
| Potassium max-min^#^ | 1.44 | 1.23-1.69 | | <0.001 | 1.16 | 0.96-1.40 | 0.117 |
| CVP max-min | 1.04 | 1.03-1.05 | | <0.001 | 1.01 | 0.99-1.03 | 0.060 |
| Cardiac index-min<2.2^##^ | 1.70 | 1.36-2.12 | | <0.001 | 1.19 | 0.94-1.51 | 0.151 |
| Sepsis | 3.17 | 2.30-4.37 | | <0.001 | 1.53 | 1.04-2.24 | **0.029** |
| IABP | 2.93 | 2.09-4.11 | | <0.001 | 1.58 | 1.04-2.39 | 0.032 |
| Vasopressin | 2.66 | 1.71-4.14 | | <0.001 | 0.64 | 0.37-1.12 | 0.119 |
| Epinephrine | 1.98 | 1.57-2.50 | | <0.001 | 1.20 | 0.91-1.57 | 0.193 |
| Norepinephrine | 2.86 | 2.16-3.79 | | <0.001 | 1.48 | 1.05-2.09 | **0.026** |
| Mechanical ventilation (12hour) | 1.25 | 1.19-1.32 | | <0.001 | 1.10 | 1.05-1.16 | **<0.001** |
| Packed red blood cell transfusion | 1.3 | 1.23-1.38 | | <0.001 | 1.14 | 1.07-1.21 | **<0.001** |

OR, Odds Ratio; CI, confidence interval; eGFR, estimated glomerular infiltration rate; CVP, central venous pressure; IABP, intra-aortic balloon pump. * referred to elective surgery. ** referred to white. ***referred to eGRF> 90 ml/min/1.73 m^2^. **** referred to coronary artery bypass grafting. # difference between maximum and minimum INR/potassium value within one month prior to hospital admission. ## minimum value of cardiac index (L/min·m^2^), and cutoff was based on minimum cardiac index value to provide sufficient oxygen. All preoperative variables, surgical information and postoperative variables were included in univariable logistic regression model. Only variables with *P*<0.05 in univariable analysis were presented in table and were entered into multivariable analysis. Variables with *P*<0.05 after adjustment were shown as bold.

Supplementary Table S6 Univariable and multivariable cox proportional hazards model of mortality following hospital discharge

| Variable | Univariable | | | Multivariable | | |
| --- | --- | --- | --- | --- | --- | --- |
|  | HR | 95%CI | *P*-value | HR | 95%CI | *P*-value |
| Age | 1.87 | 1.53-2.29 | <0.001 | 1.32 | 1.04-1.66 | 0.021 |
| Male* | 0.61 | 0.41-0.90 | 0.013 | 0.65 | 0.43-0.99 | 0.045 |
| BMI | 0.96 | 0.93-1.00 | 0.059 | 0.96 | 0.93-1.00 | 0.047 |
| eGFR** |  |  | <0.001 |  |  |  |
| 60-90 | 2.90 | 1.61-5.22 | <0.001 | 1.74 | 0.92-3.28 | 0.087 |
| <60 | 7.79 | 4.34-13.98 | <0.001 | 3.30 | 1.69-6.43 | <0.001 |
| Atrial fibrillation | 2.16 | 1.45-3.22 | <0.001 | 1.17 | 0.77-1.78 | 0.460 |
| Congestive heart failure | 3.55 | 2.41-5.25 | <0.001 | 1.62 | 1.06-2.48 | 0.027 |
| hypertension | 0.63 | 0.43-0.94 | 0.022 | 0.92 | 0.61-1.38 | 0.683 |
| Chronic liver disease | 3.66 | 1.49-9.00 | 0.005 | 3.62 | 1.43-9.18 | 0.007 |
| COPD | 4.47 | 2.07-9.62 | <0.001 | 3.15 | 1.43-6.93 | 0.004 |
| Sofa score | 1.18 | 1.10-1.27 | <0.001 | 1.05 | 0.98-1.13 | 0.174 |
| sepsis | 4.95 | 3.20-7.65 | <0.001 | 2.14 | 1.35-3.40 | 0.001 |
| IABP | 3.09 | 1.88-5.09 | <0.001 | 2.16 | 1.28-3.63 | 0.004 |
| Cardiac index-min | 2.20 | 1.32-3.66 | 0.002 | 1.31 | 0.77-2.21 | 0.319 |
| AKI^#^ |  |  | <0.001 |  |  | <0.001 |
| Early AKI | 3.40 | 2.02-5.74 | <0.001 | 2.69 | 1.57-4.61 | <0.001 |
| Late AKI | 7.98 | 4.45-14.29 | <0.001 | 4.40 | 2.42-8.02 | <0.001 |

HR, hazards ratio; CI, confidence interval; BMI, body mass index; eGFR, estimated glomerular infiltration rate; COPD, chronic obstructive pulmonary disease; IABP, intra-aortic balloon pump; AKI, acute kidney injury. * referred to female. ** referred to eGRF> 90 ml/min/1.73 m^2^. # referred to no AKI. *P*=0.75 in proportional hazards assumption.


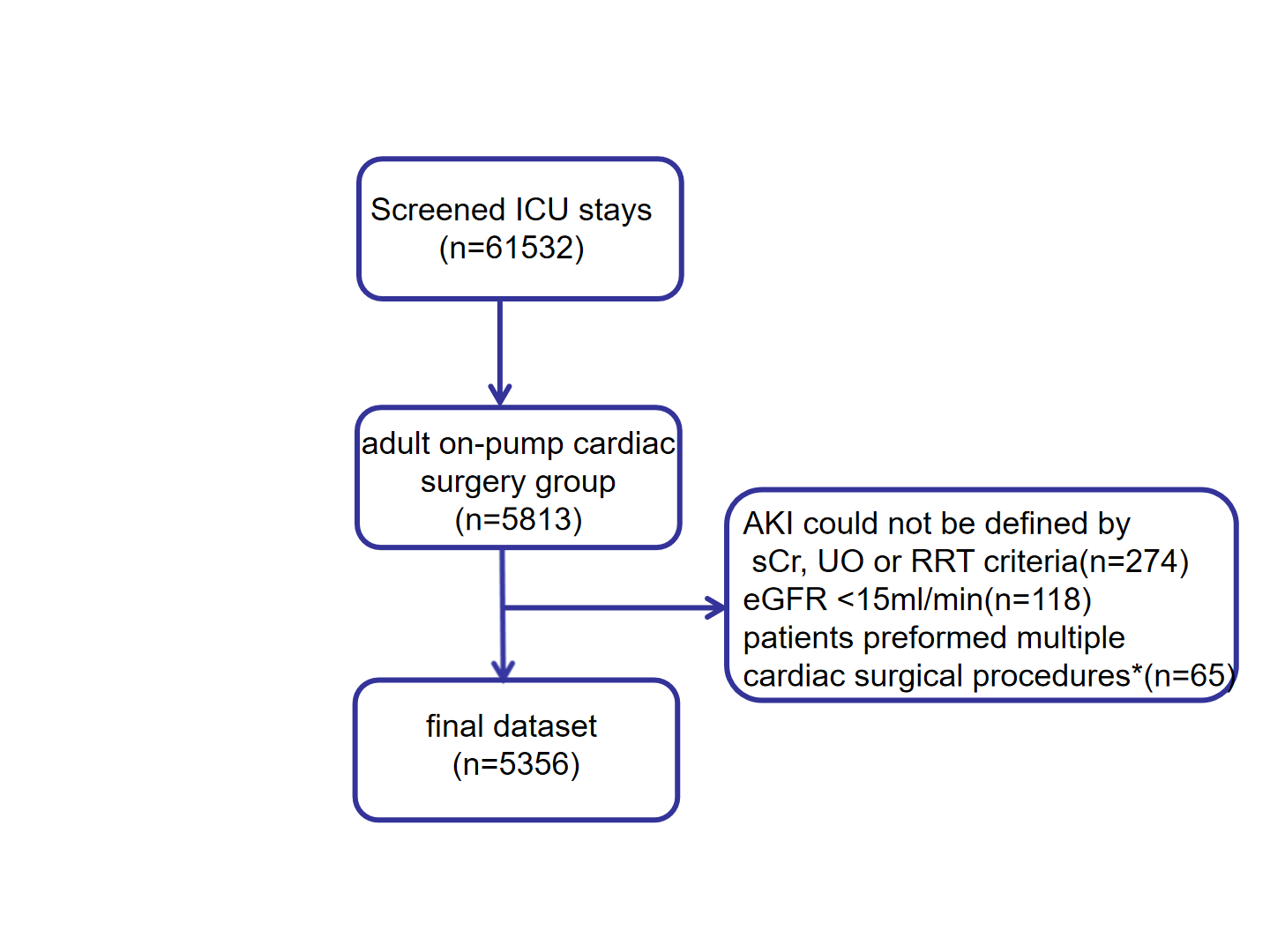


**Supplementary Figure S1** Patient flow**.** Representation of inclusive and exclusive criteria. The source of population includes all patients admitted into ICUs over a 11-year period. Since 65 patients were performed more than one cardiac surgery during the study period, we only included the first admission.


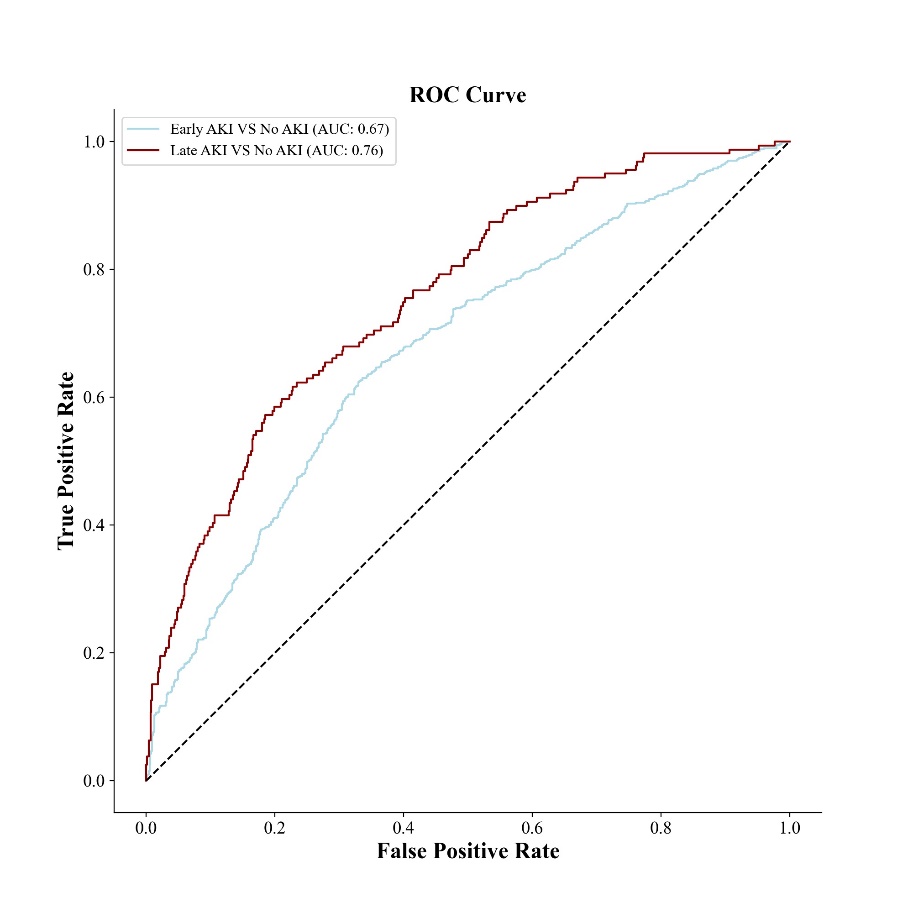


**Supplemental Figure S2.** Receiver operating characteristic curves for predicting early AKI or late AKI by random forest model. Blue line represents early AKI model, and AUROC (95%CI) was 0.67 (0.65, 0.7). Red line represents late AKI model, and AUROC (95%CI) was 0.76 (0.72, 0.79).


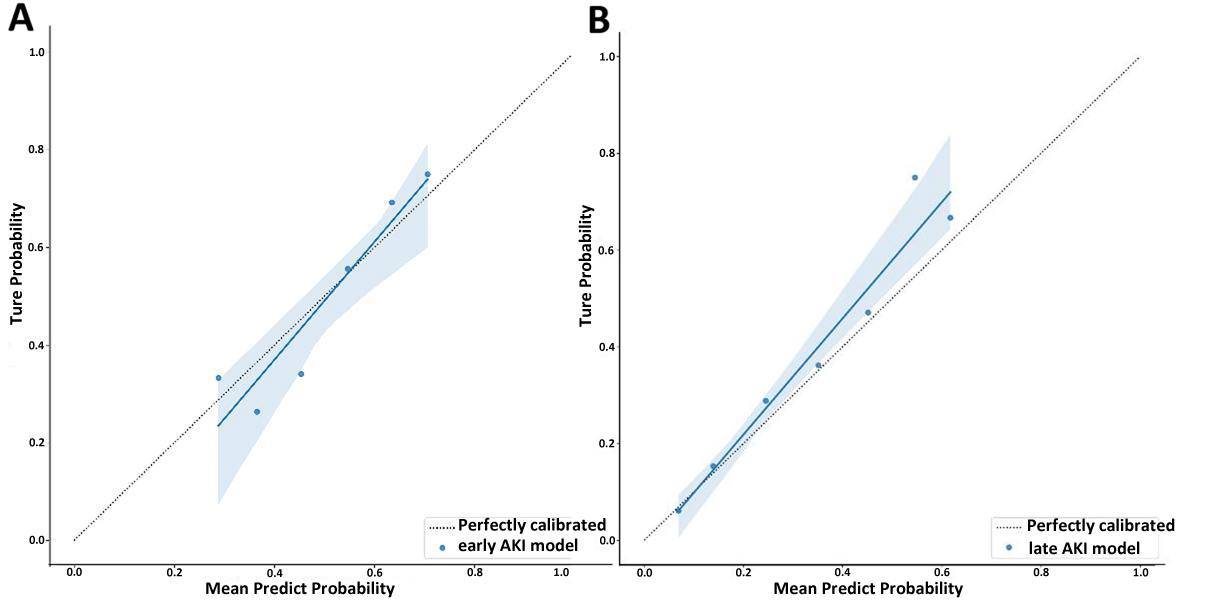


Supplementary Figure S3. Calibration curves for random forest models. Figure S3A&B were calibration curves for early AKI and late AKI models, respectively. Homser-lemeshow *p*-values for early AKI model and late AKI model were *p*<0.001.
